# Three-year Seroprevalence of SARS-CoV-2 Nucleocapsid Protein Antibody Among Children, Parental Awareness, and Contributors of Infection: A Single-school Cohort Study in Chiba, Japan

**DOI:** 10.2188/jea.JE20240284

**Published:** 2025-06-05

**Authors:** Midori Yamamoto, Kenichi Sakurai, Rieko Takatani, Aya Hisada, Chisato Mori

**Affiliations:** 1 Department of Sustainable Health Science, Center for Preventive Medical Sciences, Chiba University, Chiba, Japan; 2 Department of Nutrition and Metabolic Medicine, Center for Preventive Medical Sciences, Chiba University, Chiba, Japan; 3 Department of Clinical Medicine, Faculty of Education, Chiba University, Chiba, Japan; 4 Department of Bioenvironmental Medicine, Graduate School of Medicine, Chiba University, Chiba, Japan

**Keywords:** SARS-CoV-2, child’s infection, seroprevalence, awareness, contributing factor

## Abstract

**Background:**

Coronavirus disease 2019 (COVID-19) in children is often asymptomatic, posing challenges in detecting infections. Additionally, factors contributing to infection remain poorly understood. This study aimed to investigate trends in anti-severe acute respiratory syndrome coronavirus 2 (SARS-CoV-2) nucleocapsid antibody seroprevalence, the relationship between seroprevalence and parental perception of child infection, and factors related to COVID-19 in children.

**Methods:**

In December 2020, 355 children aged 6–12 years in one elementary school were enrolled in the study. The anti-SARS-CoV-2 nucleocapsid antibody seroprevalence was assessed, and questionnaires were administered annually for 3 years. Parents’ perceptions of infection and factors contributing to infection were examined.

**Results:**

The seroprevalence was 0.6%, 2.2%, and 60.9% in the first, second, and third years, respectively. The third-year seroprevalence among children reported as ‘infected,’ ‘not tested but had symptoms,’ and ‘not infected’ by parents was 97.3%, 83.3%, and 35.7%, respectively. Increased odds of seropositivity at the third-year measurement were observed in lower grades (adjusted odds ratio [aOR] 2.79 compared with higher grades) and in children more likely to play with others (aOR 3.97 for ‘somewhat’ and aOR 2.84 for ‘often,’ compared with ‘rarely’). No significant associations with seropositivity were found for sex, siblings, body mass index, serum 25-OH vitamin D_3_ concentration, or sleep duration.

**Conclusion:**

The Omicron variant outbreak from the end of 2021 led to a sharp increase in seroprevalence among children, with many unaware of their infection. Frequent play with others may facilitate transmission in children. These data provide useful information for developing countermeasures against COVID-19 and other future pandemics.

## INTRODUCTION

Coronavirus disease 2019 (COVID-19), an infectious disease caused by severe acute respiratory syndrome coronavirus 2 (SARS-CoV-2), was first recognized in the world towards the end of 2019.^1^^,^^2^ It caused a global pandemic, with the cumulative total number of infected people worldwide reaching 775 million in February 2024.^3^ The trends in infection rates varied widely across countries and regions.^3^^,^^4^ In November 2021, cases caused by the Omicron variant were reported, and the World Health Organization (WHO) designated it a variant of concern. Subsequently, Omicron cases have rapidly increased worldwide, becoming the predominant strain of SARS-CoV-2 infections.^5^

Since the emergence of the Omicron variant, COVID-19 cases have often been asymptomatic or mildly symptomatic.^6^ Such individuals may spread the infection while unaware of their own infection. Surveillance data based solely on diagnosed cases may underestimate the infection status, as not all COVID-19 cases have been diagnosed.^5^ A meta-analysis of studies worldwide, conducted using the standardized protocol authorized by the WHO, indicated that the estimated infectious cases with anti-SARS-CoV-2 nucleocapsid (anti-N) antibodies exceeded the number of reported cases.^5^

When COVID-19 first became widespread, children were considered less susceptible to SARS-CoV-2 infection.^7^^,^^8^ However, it has been recognized that children are also susceptible to SARS-CoV-2 but are often asymptomatic.^9^^,^^10^ In a meta-analysis of global SARS-CoV-2 seroprevalence in children from January 2020 to April 2022, the prevalence ratios compared with those among adults aged 20–29 years old were 0.75 (95% confidence interval [CI], 0.67–0.84) and 0.94 (95% CI, 0.89–1.00) for 0–9- and 10–19-year-olds, respectively.^5^ Studies in the general population and in children treated for reasons other than COVID-19 reported low seroprevalence in 2021, followed by a drastic increase in 2022.^11^^–^^16^ A cross-sectional study conducted in Latvia between March and July of 2022 reported that 173 of 200 (86.5%) hospitalized patients aged 0–18 years, who were treated for conditions other than COVID-19, tested positive for anti-N antibodies. Additionally, approximately one-third of these patients were asymptomatic and unaware of the SARS-CoV-2 infection.^17^ Because children with COVID-19 are often asymptomatic or have only a few symptoms,^18^^–^^20^ recognizing the child’s infection is difficult.^17^

A Japanese study reported that the seroprevalence of SARS-CoV-2 spike protein antibody in the Tokyo metropolitan area increased in children aged 5–9 years after the emergence of the Delta variant, which occurred in August 2021.^21^ They also reported that the seroprevalence until March 2021, which could represent the rate of infection in this area, greatly exceeded the cumulative incidence of COVID-19. This increase in seroprevalence can be explained by SARS-CoV-2 infection rather than vaccination, since SARS-CoV-2 vaccination for children aged >5 years was approved in February 2022 in Japan. Although there are few reports on the seroprevalence of anti-N antibodies in children in Japan since the outbreak of the Omicron variant, a study reported a dramatic increase in the seroprevalence of anti-N antibodies in children in the Tokyo metropolitan area and Sapporo, Japan, from December 2021 to March 2023.^22^ To our knowledge, no studies have compared parental perceptions with actual infection status since the appearance of the Omicron variant.

The risk of infection from asymptomatic COVID-19 cases has been reported to be lower than that from symptomatic cases.^19^^,^^23^ However, concerns persist that asymptomatic children may spread the infection.^24^ Understanding SARS-CoV-2 transmission among children and identifying the contributing factors are important for developing countermeasures against future COVID-19 or other pandemics. In children, the presence of comorbidities has been reported as a risk factor for severe COVID-19. Additionally, higher parental education has been reported as a risk factor for COVID-19.^25^^,^^26^ However, few reports have evaluated the relationship between COVID-19 and lifestyle and physical characteristics, especially after the Omicron variant outbreak. Therefore, we conducted a 3-year longitudinal study involving children from a single elementary school in Japan. The study aimed to clarify the following three points: 1) trends in the seroprevalence of anti-N antibodies as an indicator of infection, 2) relationships between anti-N seroprevalence and parental perception of the child’s infection, and 3) background factors related to SARS-CoV-2 infection in children.

## METHODS

### Study design and participants

**Figure 1.**
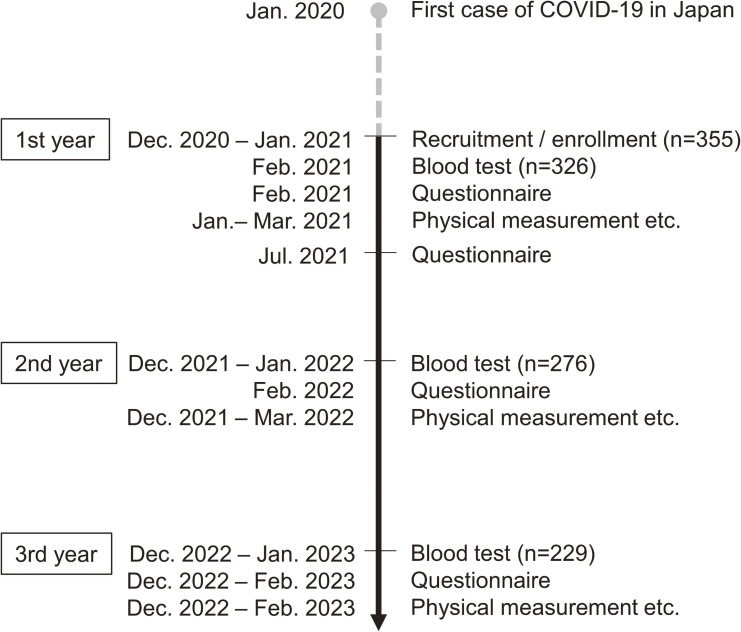
The data collection timeline in the Study of Child Health and Lifestyle during the coronavirus disease 2019 pandemic

This study utilized data from a school-based survey, the Study of Child Health and Lifestyle during the COVID-19 pandemic, a prospective cohort study that examined 1) trends in COVID-19 rates among children over time and 2) the relationship between lifestyle changes after the COVID-19 pandemic and children’s health status. In December 2020, all students at a university-affiliated elementary school in Chiba, Japan, were invited to participate. Of the 639 students at the school, 355 aged 6–12 years (grades 1–6) were enrolled (participation rate 55.6%). Blood sampling and physical measurements of children were conducted annually for 3 years: the first year, January to February 2021; the second year, December 2021 to March 2022; and the third year, December 2022 to February 2023 (Figure 1). Questionnaires completed by the children and their guardians were also administered every year. For children who graduated from elementary school in the second or third year of the survey, the survey was limited to those who agreed to continue participating. Doctors or nurses took blood samples in a multipurpose room or a gymnasium in the school. Of the 355 children who participated in the study, blood tests were conducted for 326 in the first year, 276 in the second year, and 230 in the third year of the survey, measuring serum anti-N antibodies, 25-OH vitamin D_3_, and lipids.

This study was conducted in accordance with the Declaration of Helsinki and approved by the Research Ethics Committee of the Graduate School of Medicine, Chiba University (3909). All the children and their parents who participated in the study provided written informed assent and consent, respectively. This study followed the Strengthening the Reporting of Observational Studies in Epidemiology guideline.^27^

### Serologic testing for anti-nucleocapsid protein antibodies against SARS-CoV-2

Serum samples were subjected to antibody testing using the Elecsys^®^ Anti-SARS-CoV-2 assay on the Cobas^®^ 8000 e801 module (Roche Diagnostics, Rotkreuz, Switzerland). This test is an electrochemiluminescence immunoassay for in vitro qualitative detection of antibodies (IgM/IgG) against the SARS-CoV-2 nucleocapsid protein in human serum. A cut-off index of ≥1.0 was considered positive for anti-N antibodies. The specificity and sensitivity at ≥14 days after polymerase chain reaction confirmation were reported to be 99.80% (95% CI, 99.69–99.88%) and 99.5% (95% CI, 97.0–100.0%), respectively.^28^

### Parental awareness of children’s SARS-CoV-2 infection

Parental awareness of SARS-CoV-2 infection in their children was investigated using a self-reported questionnaire administered from January to February 2023. The question ‘Has your child been infected with the SARS-CoV-2?’ was answered with the following options: ‘Not infected,’ ‘Not tested but had symptoms,’ and ‘Infected.’ In the case of the latter two responses, the respondents were asked when their child became infected or showed symptoms. Cases where parents indicated their children were infected after the third year of antibody testing were excluded.

### Possible factors associated with SARS-CoV-2 infection in children

The following variables were used to explore the possible factors related to SARS-CoV-2 infection in the third year of the study: school grade, sex, and presence of sibling(s). In addition, the following data from physical measurements, blood tests, and questionnaires in the second year of the study were used as possible factors related to immunity: body mass index (BMI), serum 25-OH vitamin D_3_, sleeping hours at night, tendency to play with others, and SARS-CoV-2 vaccination (Table 1). Age- and sex-specific percentiles of BMI were calculated using the reference data for Japanese children.^29^ Serum 25-OH vitamin D_3_ levels were measured using liquid chromatography–tandem mass spectrometry.^30^ For sleep duration, parents were asked about their children’s regular bedtimes and wake-up times on weekdays and weekends, and the average daily sleep duration was calculated for five weekdays and two holidays per week. Regarding the tendency to play with others, parents were asked to what extent the following statement applied to their children: ‘Rather prefer to be alone and often play alone.’ Responses were assigned from the following three categories: not true (often [to play with others]), somewhat true (somewhat), and certainly true (rarely).

**Table 1.** Descriptive statistics for basic characteristics and possible factors associated with SARS-CoV-2 infection

	Lower grades^a^		Higher grades^a^		*P*-value^c^
	*N*	(%)^b^	*N*	(%)^b^	
All children	184		171		
Grade at enrolment (range)	1–3		4–6		
Age at enrolment, years (range)	6–9		9–12		
Girls	96	(52.2)	85	(49.7)	0.672
Sibling(s)					
None	77	(41.8)	59	(34.5)	0.292
1	75	(40.8)	83	(48.5)	
≥2	32	(17.4)	29	(17.0)	

*Data at the second-year survey*					
Participating children	184		153		
Body mass index					
<15th percentile	41	(22.3)	29	(19.0)	0.503
15th to 85th percentile	117	(63.6)	93	(60.8)	
≥85th percentile	25	(13.6)	13	(8.5)	
Missing	1	(0.5)	18	(11.8)	
Serum 25-OH vitamin D_3_					
<20 ng/mL	77	(41.8)	75	(49.0)	0.092
≥20 ng/mL	79	(42.9)	50	(32.7)	
Missing	28	(15.2)	28	(18.3)	
Sleeping hours at night					
<8 hours/day	4	(2.2)	10	(6.5)	0.026
≥8 hours/day	173	(94.0)	116	(75.8)	
Missing	7	(3.8)	27	(17.6)	
Playing with others					
rarely	20	(10.9)	19	(12.4)	0.671
somewhat	57	(31.0)	40	(26.1)	
often	89	(48.4)	62	(40.5)	
missing	18	(9.8)	32	(20.9)	
Vaccination (until the second year)^d^					
None	—		79	(51.6)	—
1 dose	—		3	(2.0)	
2 doses	—		45	(29.4)	
Missing	—		26	(17.0)	

*Data until the third-year survey*					
Participating children	183		128		
Vaccination (until the third year)					
None	105	(57.1)	38	(29.7)	<0.001
1 dose	2	(1.1)	2	(1.6)	
2 doses	41	(22.3)	16	(12.5)	
3 doses	23	(12.5)	40	(31.3)	
Missing	12	(6.6)	32	(25.0)	

SARS-CoV-2, severe acute respiratory syndrome coronavirus 2.

^a^Lower grades were elementary school grades 1–3, and higher grades were elementary school grades 4–6 in the first year.

^b^Percentage was calculated based on the number of participating children of the year.

^c^*P* values for differences among grades were estimated using Fisher’s exact test or Pearson’s chi-square test.

^d^Vaccination was not available for children under 12 years of age.

### Statistical analysis

The following four analyses were conducted: 1) Descriptive statistics were performed for each category of basic characteristics and possible infection factors in children in grades 1–3 and 4–6. Pearson’s chi-square test or Fisher’s exact test was used to test for between-group differences. 2) Major characteristics of children were examined by school grade. 3) To determine the relationship between actual SARS-CoV-2 infection in children and parental awareness, the prevalence of anti-N antibodies in the third-year measurements was calculated for each level of parental awareness of their child’s infection. 4) To explore the factors associated with SARS-CoV-2 infection in the third year, a multivariable binomial logistic regression was performed on children who were negative for anti-N antibodies at the second-year measurements. The odds ratios (ORs) and 95% CIs for positive anti-N antibody results were calculated using the aforementioned possible factors of infection as independent variables. All analyses were performed using SPSS, version 27 (IBM Corp., Armonk, NY, USA). The COVID-19 positivity rates in Japan and Chiba Prefecture were calculated by dividing the monthly number of newly confirmed cases by the total population estimates each year. We used COVID-19 trend data from the Ministry of Health, Labour and Welfare based on reports from each municipality for the former^31^ and census data for the latter.^32^

## RESULTS

### Characteristics of the study population

Table 1 presents the basic characteristics of the children, stratified into lower and higher grades. The lower grades (grades 1–3, aged 6–9 years) consisted of 184 children and the higher grades (grades 4–6, aged 9–12 years) comprised 171 children in the first year of the study, with 52.2% and 49.7% of the children being girls, respectively. Of the higher-grade children in the second year, 29.4% reported having received two doses of SARS-CoV-2 vaccination. Children under 12 years of age were not eligible for vaccination then. In the third year, 22.3% and 12.5% of lower-grade children received two and three doses, respectively, and 12.5% and 31.3% of higher-grade children received two and three doses, respectively. The only vaccine available for children was Pfizer-BioNTech’s mRNA BNT162b2 vaccine.

Table 2 shows the major characteristics of the children from each grade. Dropout rates were 5% in the second year and 12% in the third year, mainly due to elementary school graduation. Decreased response rates of the guardians to the questionnaire were observed in the higher grades group (73–92% in the second year and 75–84% in the third year).

**Table 2.** Major characteristics of children in each grade

		Lower grades			Higher grades			Total
School grade								
first year		E1	E2	E3	E4	E5	E6	
second year		E2	E3	E4	E5	E6	JH1	
third year		E3	E4	E5	E6	JH1	JH2	
Age, years								
first year		6–7	7–8	8–9	9–10	10–11	11–12	
second year		7–8	8–9	9–10	10–11	11–12	12–13	
third year		8–9	9–10	10–11	11–12	12–13	13–14	
Number of participating children								
first year		51	64	69	68	49	54	355
second year		51	64	69	68	48	37	337
third year		50	64	69	65	26	37	311
Dropout rates								
second year		0%	0%	0%	0%	2%	31%	5%
third year		2%	0%	0%	4%	47%	31%	12%
Response rates of guardians’ questionnaire^a^								
first year		100%	100%	100%	99%	100%	100%	100%
second year		96%	97%	99%	85%	92%	73%	91%
third year		98%	95%	94%	75%	77%	84%	88%
Number of seropositive children/number of blood tests								
first year		0/39	0/59	0/66	0/64	2/47	0/51	2/326
second year		1/39	1/58	1/57	0/60	3/40	0/22	6/276
third year		27/40	34/49	39/57	21/44	7/15	12/25	140/230
Seropositivity, % (95% CI)^b^								
first year		0	0	0	0	4.3	0	0.6
		(0–0)	(0–0)	(0–0)	(0–0)	(0–10.2)	(0–0)	(0–1.5)
second year		2.6	1.7	1.8	0	7.5	0	2.2
		(0–7.8)	(0–5.2)	(0–5.3)	(0–0)	(0–16.0)	(0–0)	(0.4–3.9)
third year		67.5	69.4	68.4	47.7	46.7	48.0	60.9
		(52.3–82.7)	(56.0–82.8)	(56.0–80.9)	(32.4–63.1)	(18.1–75.3)	(27.0–69.0)	(54.5–67.2)
Vaccination rates^a^								
second year^c^	None	—	—	—	—	38%	8%	—
	1 dose	—	—	—	—	6%	0%	—
	2 doses	—	—	—	—	48%	59%	—
	missing	—	—	—	—	8%	32%	—
third year	None	62%	69%	43%	48%	15%	8%	46%
	1 dose	0%	2%	1%	3%	0%	0%	1%
	2 doses	26%	14%	28%	14%	8%	14%	18%
	3 doses	6%	8%	22%	8%	54%	57%	20%
	missing	6%	8%	6%	28%	23%	22%	14%

CI, confidence interval; E1 to E6, elementary school grade 1 to grade 6; JH1 and JH2, junior high-school grade 1 and grade 2.

^a^Percentage per participating children of the year.

^b^Percentage per children who received blood test.

^c^Vaccination was not available for children under 12 years of age.

### Positive rates of SARS-CoV-2 nucleocapsid protein antibodies over 3 years

As shown in Table 2, the prevalence of anti-N antibodies for all grades was 0.6% at the first-year measurement (January 2021), 2.2% at the second-year measurement (December 2021 to January 2022), and 60.9% at the third-year measurement (December 2022 to January 2023). Only two children who tested positive at the first-year measurement were negative at the second-year measurement. No child tested positive at the second-year measurement and negative at the third-year measurement. Seroprevalence in the third year was 67.5–69.4% in the lower grades (elementary school grades 3–5 in the third year) and 46.7–48.0% in the higher grades (elementary school grade 6 to junior high school grade 2 in the third year).

### Parental perception of children’s infection and seroprevalence of SARS-CoV-2 nucleocapsid protein antibodies

**Figure 2.**
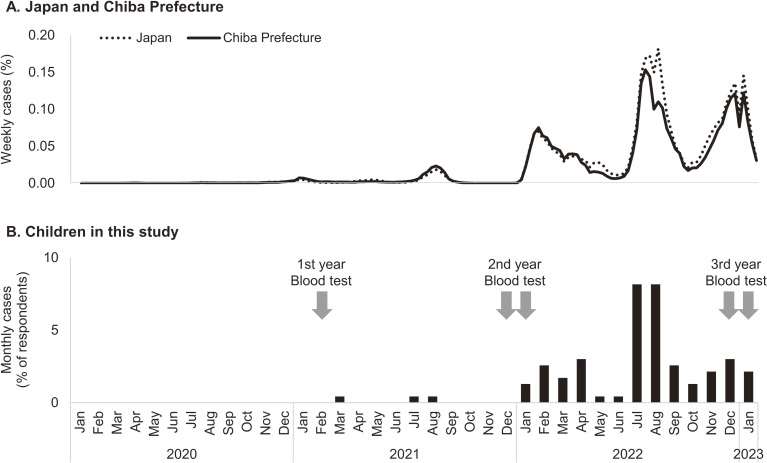
Reported cases of SARS-CoV-2 infection (%). (**A**) In all of Japan and Chiba Prefecture and (**B**) in children whose parents responded to the questionnaire in this study. SARS-CoV-2, severe acute respiratory syndrome coronavirus 2.

Table 3 presents the prevalence of anti-N antibodies according to the parental perception of their child’s infection in the third year of the study. Of the 208 children who underwent antibody testing and whose parents responded to the questions, 55.3% indicated that their children had not been infected with SARS-CoV-2, 8.7% indicated that their children had not been tested but had symptoms, and 36.1% indicated that their children had been infected with SARS-CoV-2. The seroprevalence in the groups in which parents reported that their children were ‘infected’ and ‘not tested but had symptoms’ was 97.3% (95% CI, 93.6–100.0%) and 83.3% (95% CI, 64.3–100.0%), respectively. On the other hand, 35.7% (95% CI, 26.8–44.5%) of the ‘not infected’ group were also positive. Of the seropositive children, 31.8% were asymptomatic and unaware of their infection, and 11.6% were not tested for symptoms suggestive of COVID-19. Figure 2 illustrates the timing of infection in the children as reported by their parents and the timing of blood tests. The SARS-CoV-2 infection trends among children in this cohort were almost consistent with those in Japan and Chiba Prefecture.

**Table 3.** Parental perception of child’s infection and seroprevalence of SARS-CoV-2 nucleocapsid protein antibodies at the third year

Parental perception of child’s infection	Total		SARS-CoV-2 nucleocapsid protein antibody					

			Positive		Negative		Crude seroprevalence	
	*n*	(%)	*n*	(%)	*n*	(%)	%	(95% CI)
Not infected	115	(55.3)	41	(31.8)	74	(93.7)	35.7	(26.8–44.5)
Not tested but had symptoms	18	(8.7)	15	(11.6)	3	(3.8)	83.3	(64.3–100.0)
Infected	75	(36.1)	73	(56.6)	2	(2.5)	97.3	(93.6–100.0)
Total	208	(100.0)	129	(100.0)	79	(100.0)	62.0	(55.4–68.7)

CI, confidence interval; SARS-CoV-2, severe acute respiratory syndrome coronavirus 2.

### Factors associated with SARS-CoV-2 nucleocapsid protein antibody positivity in the third year

Table 4 shows the results of a multivariable logistic regression analysis performed on children who were seronegative for anti-N antibodies in the second-year measurement. Increased odds of anti-N antibody seropositivity at the third-year measurement were observed in lower grades (adjusted OR [aOR] 2.79; 95% CI, 1.32–5.93 compared with higher grades) and in children more likely to play with others (aOR 3.97; 95% CI, 1.31–12.02 for ‘somewhat’ and aOR 2.84; 95% CI, 1.00–8.09 for ‘often,’ compared with ‘rarely’). Children vaccinated before the second-year survey had lower ORs for seropositivity (aOR 0.49; 95% CI, 0.14–1.71 for two doses), whereas children with more siblings had higher ORs for seropositivity (aOR 1.30; 95% CI, 0.64–2.66 for one sibling and aOR 2.37; 95% CI, 0.85–6.55 for two or more siblings); however, these associations were not significant. In addition, no significant associations with seropositivity were found for sex, BMI, serum 25-OH vitamin D_3_ concentration, or sleeping hours.

**Table 4.** Association of child’s factors with the presence of SARS-CoV-2 nucleocapsid protein antibodies at the third-year examination in children who were negative in the second year (*n* = 179)

Factors	*N*	cOR	(95% CI)	aOR	(95% CI)
Lower grades^a^ (reference: higher grades^a^)	117	**2.84**	**(1.50–5.38)**	**2.79**	**(1.32–5.93)**
Girls (reference: boys)	93	1.08	(0.59–1.98)	0.92	(0.47–1.79)
Sibling(s) (reference: none)					
1	77	1.37	(0.72–2.64)	1.30	(0.64–2.66)
≥2	27	1.87	(0.73–4.79)	2.37	(0.85–6.55)
*Data at the second year*					
Body mass index (reference: 15th to 85th percentile)					
<15th percentile	40	1.39	(0.65–2.98)	1.65	(0.71–3.81)
≥85th percentile	22	0.97	(0.38–2.45)	1.02	(0.37–2.79)
Serum 25-OH vitamin D_3_, <20 ng/mL (reference: ≥20 ng/mL)	91	1.11	(0.61–2.02)	1.36	(0.70–2.64)
Sleeping hours at night, <8 hours/day (reference: ≥8 hours/day)	6	0.62	(0.12–3.15)	0.73	(0.12–4.56)
Playing with others (reference: rarely)					
somewhat	65	**2.82**	**(1.05–7.62)**	**3.97**	**(1.31–12.02)**
often	92	2.46	(0.95–6.37)	**2.84**	**(1.00–8.09)**
Vaccination (reference: none)					
1 dose	1	NA		NA	
2 doses	16	**0.25**	**(0.08–0.75)**	0.49	(0.14–1.71)

aOR, adjusted odds ratio; CI, confidence interval; cOR, crude odds ratio; SARS-CoV-2, severe acute respiratory syndrome coronavirus 2; NA, not available due to only one cases.

^a^Lower grades were grades 3–5, and higher grades were grades 6–8 in the third year.

## DISCUSSION

In this study, we evaluated the changes in SARS-CoV-2 infection in Japanese children over a period of 3 years, up to January 2023. We also compared the relationship between the children’s infections and their parents’ awareness and explored the factors associated with infection. The results revealed the following: First, the number of SARS-CoV-2 infections detected through seropositivity for anti-N antibodies was low until January 2022. However, the number of seropositive cases increased in the third-year survey conducted from December 2022 to January 2023. Second, >40% of the seropositive patients were unaware of their infection or had not been tested for COVID-19 despite having symptoms. Third, the increased seropositivity for anti-N antibodies was associated with lower-grade students and a tendency to play with others. The seroprevalence of anti-N antibodies increased sharply in the third year of measurements (December 2022 to January 2023). This result is consistent with the increase in the number of SARS-CoV-2-infected children observed in the parents’ responses to the current survey. A similar situation was observed across the elementary school surveyed in this study, where the number of SARS-CoV-2-infected children increased in 2022. Similarly, the number of SARS-CoV-2 cases reported in Chiba Prefecture and Japan increased in three waves from early 2022 to early 2023.^31^^,^^32^ In line with this, an increase in the seroprevalence of anti-N antibodies from December 2021 to March 2023 (from 4% to 23%) was reported in all age groups in the Tokyo metropolitan area, Japan, with a particularly marked increase among young people.^22^ The increase may be explained by the emergence of the Omicron variant, which was designated a ‘variant of concern’ by WHO in November 2021 and became predominant globally in January 2022.^5^^,^^16^^,^^33^ This variant became predominant in Japan, including Chiba Prefecture, during this period.^34^ The Omicron variant is more infectious to children as well as adults.^35^^,^^36^ This could be attributed to its higher transmissibility, owing to multiple mutations in the spike protein.^37^ Other factors contributing to the increase may include the gradual decrease in restrictions on school life activities in Japan and the removal of masks to prevent heat stroke in the summer.^38^

Several studies in other countries have also reported a rapid increase in the number of SARS-CoV-2 cases in children and adults since the outbreak of the Omicron variant.^11^^–^^16^ Additionally, many cases have been reported in individuals who had few or no symptoms and were unaware of their SARS-CoV-2 infection.^11^^,^^12^^,^^17^ The present study showed that the situation was similar in Japan. This finding, which indicates that many children may unknowingly spread the infection in their daily lives, provides evidence for future infection control measures.

The present study provides new insights into SARS-CoV-2 infection in children, as lower grades (age) and a tendency to play with others were associated with seropositive conversion from the second to the third survey. One possible reason for lower-grade students being more susceptible to SARS-CoV-2 infection is their low vaccination coverage. In Japan, conventional vaccination began in June 2021 for people aged ≥12 years. Later, vaccination for children aged 5–11 years was initiated in February 2022. Systematic reviews have shown that booster vaccination with conventional vaccines effectively protects against the Omicron variant.^39^^,^^40^ Therefore, the fact that more children in the upper grades received the booster vaccine in the second year of the study may have contributed to the lower seroprevalence. Another possible reason is that lower-grade students tend to communicate more closely with each other than the upper-grade students.^41^^,^^42^ Additionally, it is difficult to thoroughly implement infection control measures, such as social distancing and wearing masks, because self-regulation skills are developed from early childhood through the early school years.^43^

Our study found that a tendency to play with others was associated with increased seropositivity. This indicates that more contact with others at school or after school presents a higher risk for SARS-CoV-2 transmission. In Japan, despite concerns about the spread of infections and an increase in the number of infected children after the outbreak of the Omicron variant, the policy of wearing masks outdoors and movement restrictions have been relaxed to prevent heat stroke and for educational considerations. Children are prone to frequent physical contact and often stay in close proximity to other children. The increase in the number of SARS-CoV-2-infected children in this study, especially in the summer of 2022, suggests that the emergence of the more infectious Omicron variant, unmasking, and increased contact with other children may have contributed to the spread of infection. Nevertheless, playing with each other is beneficial to the physical and mental health of children. Even though this study suggests that contact with other children is a major factor in the transmission of SARS-CoV-2, children should not be restricted from playing together. Outdoor transmission of SARS-CoV-2 has been shown to be dramatically lower than indoor transmission, due to airflow, ventilation, and ultraviolet radiation from sunlight,^44^ so encouraging children to play outdoors rather than indoors may help control the spread of infection.

### Strengths and limitations

The strength of this study lies in our ability to accurately assess the actual infection status by monitoring anti-N antibodies in all grades of one school over a 3-year period. Second, we collected information on parents’ perceptions and children’s lifestyles and assessed the associations among these parameters.

This study had several limitations. First, the participation rate in this cohort study was 55.6%. Nonparticipation may have been mainly due to the child’s refusal to provide blood samples; thus, there may have been little to no selection bias in seroprevalence. Many upper-grade students did not continue their participation in the study after graduation (retention rate, 33.3%), thereby increasing the missing values. Second, some seronegative children may include those who were infected but had a weak immune response to SARS-CoV-2 or reduced antibodies. In our study, two seropositive children at the first-year measurement were seronegative at the second-year measurement. The antibody dynamics after SARS-CoV-2 infection varies widely between individuals, with children and asymptomatic patients tending to have lower antibody levels.^45^^–^^48^ For IgG levels, which persist longer than IgM, a significant decline has been reported in children up to 180 days after infection.^46^ Thus, uncertainty remains regarding the infection rate, which may be slightly higher. Third, some information, such as vaccination and COVID-19 status, was collected through parental responses, which may be subject to recall bias. Fourth, this study did not examine the frequency of contact with other children, such as time spent playing together or the number of children they played with. Instead, we collected data on the child’s tendency to play with others, which may not accurately indicate the actual frequency of contact with other children. Fifth, the result that children with more siblings tended to be at a higher risk of infection suggests transmission among siblings. However, as this study did not investigate household transmission, we could not compare the risk of infection at home with that at school or other settings. Finally, given that our results were based on a survey of a single-school cohort, generalizations should be made with caution.

### Conclusion

We evaluated the 3-year SARS-CoV-2 infection trend in school-aged Japanese children using antibody testing. SARS-CoV-2 infection in children increased after the emergence of the Omicron variant, and in nearly half of them, parents were unaware of their children’s infections. Playing with other children and young age were associated with infections. These data provide useful information for developing countermeasures against future COVID-19 outbreak and other pandemics. Future studies should investigate how transmission of different SARS-CoV-2 variants and infection control measures affect the course of a pandemic in the context of widespread vaccination.

## References

[1] WHO. Origin of SARS-CoV-2. https://iris.who.int/bitstream/handle/10665/332197/WHO-2019-nCoV-FAQ-Virus_origin-2020.1-eng.pdf; 2020 Accessed 06.03.2024.

[2] Li Q, Guan X, Wu P, . Early transmission dynamics in Wuhan, China, of novel coronavirus-infected pneumonia. N Engl J Med. 2020;382:1199–1207. 10.1056/NEJMoa2001316 31995857 PMC7121484

[3] WHO. COVID-19 dashboard. https://data.who.int/dashboards/covid19/cases?n=c; 2024 Accessed 06.03.2024.

[4] COVID-19 Cumulative Infection Collaborators. Estimating global, regional, and national daily and cumulative infections with SARS-CoV-2 through Nov 14, 2021: a statistical analysis. Lancet. 2022;399:2351–2380. 10.1016/S0140-6736(22)00484-6 35405084 PMC8993157

[5] Bergeri I, Whelan MG, Ware H, ; Unity Studies Collaborator Group. Global SARS-CoV-2 seroprevalence from January 2020 to April 2022: a systematic review and meta-analysis of standardized population-based studies. PLoS Med. 2022;19:e1004107. 10.1371/journal.pmed.1004107 36355774 PMC9648705

[6] Joung SY, Ebinger JE, Sun N, . Awareness of SARS-CoV-2 Omicron variant infection among adults with recent COVID-19 seropositivity. JAMA Netw Open. 2022;5:e2227241. 10.1001/jamanetworkopen.2022.27241 35976645 PMC9386542

[7] Lai CC, Wang JH, Hsueh PR. Population-based seroprevalence surveys of anti-SARS-CoV-2 antibody: an up-to-date review. Int J Infect Dis. 2020;101:314–322. 10.1016/j.ijid.2020.10.011 33045429 PMC7546669

[8] Viner RM, Mytton OT, Bonell C, . Susceptibility to SARS-CoV-2 infection among children and adolescents compared with adults: a systematic review and meta-analysis. JAMA Pediatr. 2021;175:143–156. 10.1001/jamapediatrics.2020.4573 32975552 PMC7519436

[9] Levorson RE, Christian E, Hunter B, . A cross-sectional investigation of SARS-CoV-2 seroprevalence and associated risk factors in children and adolescents in the United States. PLoS One. 2021;16:e0259823. 10.1371/journal.pone.0259823 34748615 PMC8575286

[10] Zinszer K, McKinnon B, Bourque N, . Seroprevalence of SARS-CoV-2 antibodies among children in school and day care in Montreal, Canada. JAMA Netw Open. 2021;4:e2135975. 10.1001/jamanetworkopen.2021.35975 34812845 PMC8611475

[11] Engels G, Oechsle AL, Schlegtendal A, ; IMMUNEBRIDGE KIDS study group. SARS-CoV-2 sero-immunity and quality of life in children and adolescents in relation to infections and vaccinations: the IMMUNEBRIDGE KIDS cross-sectional study, 2022. Infection. 2023;51:1531–1539. 10.1007/s15010-023-02052-5 37280412 PMC10243264

[12] Franczak J, Moppert J, Sobolewska-Pilarczyk M, Pawłowska M. The seroprevalence of SARS-CoV-2 IgG antibodies in children hospitalized for reasons other than COVID-19. J Clin Med. 2022;11:3819. 10.3390/jcm11133819 35807103 PMC9267741

[13] Filippatos F, Tatsi EB, Dellis C, . SARS-CoV-2 seroepidemiology in paediatric population during Delta and Omicron predominance. Epidemiol Infect. 2022;150:e177. 10.1017/S0950268822001601 36345855 PMC9671918

[14] Ott R, Achenbach P, Ewald DA, . SARS-CoV-2 seroprevalence in preschool and school-age children—population screening findings from January 2020 to June 2022. Dtsch Arztebl Int. 2022;119:765–770. 36345616 10.3238/arztebl.m2022.0355 PMC9884841

[15] Skowronski DM, Kaweski SE, Irvine MA, . Serial cross-sectional estimation of vaccine- and infection-induced SARS-CoV-2 seroprevalence in British Columbia, Canada. CMAJ. 2022;194:E1599–E1609. 10.1503/cmaj.221335 36507788 PMC9828974

[16] Suntronwong N, Vichaiwattana P, Klinfueng S, . SARS-CoV-2 infection-induced seroprevalence among children and associated risk factors during the pre- and omicron-dominant wave, from January 2021 through December 2022, Thailand: a longitudinal study. PLoS One. 2023;18:e0279147. 10.1371/journal.pone.0279147 37104299 PMC10138857

[17] Sapronova K, Kaķe R, Pavāre J, . SARS-CoV-2 seroprevalence among children in Latvia: a cross-sectional study. Medicine (Baltimore). 2023;102:e32795. 10.1097/MD.0000000000032795 36820593 PMC9907906

[18] Bi Q, Wu Y, Mei S, . Epidemiology and transmission of COVID-19 in 391 cases and 1286 of their close contacts in Shenzhen, China: a retrospective cohort study. Lancet Infect Dis. 2020;20:911–919. 10.1016/S1473-3099(20)30287-5 32353347 PMC7185944

[19] Chung E, Chow EJ, Wilcox NC, . Comparison of symptoms and RNA levels in children and adults with SARS-CoV-2 infection in the community setting. JAMA Pediatr. 2021;175:e212025. 10.1001/jamapediatrics.2021.2025 34115094 PMC8491103

[20] Schonfeld D, Fernández H, Ramírez J, . SARS-CoV-2 seroprevalence in the city of Puerto Madryn: underdiagnosis and relevance of children in the pandemic. PLoS One. 2022;17:e0263679. 10.1371/journal.pone.0263679 35286328 PMC8920177

[21] Yamayoshi S, Iwatsuki-Horimoto K, Okuda M, . Age-stratified seroprevalence of SARS-CoV-2 antibodies before and during the vaccination era, Japan, February 2020–March 2022. Emerg Infect Dis. 2022;28:2198–2205. 10.3201/eid2811.221127 36198306 PMC9622230

[22] Yamayoshi S, Nagai E, Mitamura K, . Seroprevalence of severe acute respiratory syndrome coronavirus 2 N antibodies between December 2021 and march 2023 in Japan. Epidemiol Infect. 2024;152:e24. 10.1017/S0950268824000141 38258464 PMC10894890

[23] Li F, Li YY, Liu MJ, . Household transmission of SARS-CoV-2 and risk factors for susceptibility and infectivity in Wuhan: a retrospective observational study. Lancet Infect Dis. 2021;21:617–628. 10.1016/S1473-3099(20)30981-6 33476567 PMC7833912

[24] Han MS, Choi EH, Chang SH, . Clinical characteristics and viral RNA detection in children with coronavirus disease 2019 in the Republic of Korea. JAMA Pediatr. 2021;175:73–80. 10.1001/jamapediatrics.2020.3988 32857112 PMC7455883

[25] Lee KS, Kim YK, Choi YY, Choe YJ, Kim MH, Lee H. Risk factors for severe and critical coronavirus disease 2019 in children. Pediatr Infect Dis J. 2024;43:234–241. 10.1097/INF.0000000000004193 38241652

[26] Wypych-Ślusarska A, Krupa-Kotara K, Oleksiuk K, Głogowska-Ligus J, Słowiński J, Niewiadomska E. Socioeconomic and health determinants of the prevalence of COVID-19 in a population of children with respiratory diseases and symptoms. Children (Basel). 2024;11:88. 10.3390/children11010088 38255401 PMC10814875

[27] Vandenbroucke JP, von Elm E, Altman DG, ; STROBE Initiative. Strengthening the Reporting of Observational Studies in Epidemiology (STROBE): explanation and elaboration. PLoS Med. 2007;4:e297. 10.1371/journal.pmed.0040297 17941715 PMC2020496

[28] Muench P, Jochum S, Wenderoth V, . Development and validation of the Elecsys Anti-SARS-CoV-2 immunoassay as a highly specific tool for determining past exposure to SARS-CoV-2. J Clin Microbiol. 2020;58:e01694-20. 10.1128/JCM.01694-20 32747400 PMC7512151

[29] Kato N, Takimoto H, Sudo N. The cubic functions for spline smoothed L, S and M values for BMI reference data of Japanese children. Clin Pediatr Endocrinol. 2011;20:47–49. 10.1297/cpe.20.47 23926394 PMC3687634

[30] Mochizuki A, Kodera Y, Saito T, . Preanalytical evaluation of serum 25-hydroxyvitamin D3 and 25-hydroxyvitamin D2 measurements using LC-MS/MS. Clin Chim Acta. 2013;420:114–120. 10.1016/j.cca.2012.10.041 23123828

[31] Ministry of Health, Labour and Welfare. Visualizing the data: information on COVID-19 infections. https://covid19.mhlw.go.jp/en/; 2023 Accessed 06.03.2024.

[32] Japanese Government. Portal site of official statistics of Japan, population of estimates. https://www.e-stat.go.jp/en/stat-search/files?page=1&toukei=00200524&metadata=1&data=1; 2023 Accessed 30.05.2024.

[33] WHO. Enhancing response to Omicron SARS-CoV-2 variant: Technical brief and priority actions for Member States. https://www.who.int/publications/m/item/enhancing-readiness-for-omicron-(b.1.1.529)-technical-brief-and-priority-actions-for-member-states; 2022 Accessed 30.05.2024.

[34] National Institute of Infectious Diseases. [Detection of SARS-CoV-2 by strain through genomic surveillance, domestic PANGO lineage changes for SARS-CoV-2 genomes (as of 28/01/2022).] (in Japanese) https://www.niid.go.jp/niid/images/cepr/covid-19/220202_genome_surveillance.pdf; 2022 Accessed 30.05.2024.

[35] Chun JY, Jeong H, Kim Y. Identifying susceptibility of children and adolescents to the Omicron variant (B.1.1.529). BMC Med. 2022;20:451. 10.1186/s12916-022-02655-z 36419108 PMC9684890

[36] Elliott P, Eales O, Steyn N, . Twin peaks: the Omicron SARS-CoV-2 BA.1 and BA.2 epidemics in England. Science. 2022;376:eabq4411. 10.1126/science.abq4411 35608440 PMC9161371

[37] Araf Y, Akter F, Tang YD, . Omicron variant of SARS-CoV-2: genomics, transmissibility, and responses to current COVID-19 vaccines. J Med Virol. 2022;94:1825–1832. 10.1002/jmv.27588 35023191 PMC9015557

[38] Ministry of the Environment, Ministry of Health Labour and Welfare. Remove your mask outdoors to prevent heatstroke. https://www.niph.go.jp/h-crisis/wp-content/uploads/2022/09/20220829104143_content_000981003.pdf; 2022 Accessed 06.03.2024.

[39] Chenchula S, Karunakaran P, Sharma S, Chavan M. Current evidence on efficacy of COVID-19 booster dose vaccination against the Omicron variant: a systematic review. J Med Virol. 2022;94:2969–2976. 10.1002/jmv.27697 35246846 PMC9088621

[40] Zou Y, Huang D, Jiang Q, Guo Y, Chen C. The vaccine efficacy against the SARS-CoV-2 Omicron: a systemic review and meta-analysis. Front Public Health. 2022;10:940956. 10.3389/fpubh.2022.940956 35910897 PMC9326247

[41] Aiello JR, De Carlo Aiello T. The development of personal space: proxemic behavior of children 6 through 16. Hum Ecol Interdiscip J. 1974;2:177–189. 10.1007/BF01531420

[42] Burgess JW. Development of social spacing in normal and mentally retarded children. J Nonverbal Behav. 1981;6:89–95. 10.1007/BF00987284

[43] Montroy JJ, Bowles RP, Skibbe LE, McClelland MM, Morrison FJ. The development of self-regulation across early childhood. Dev Psychol. 2016;52:1744–1762. 10.1037/dev0000159 27709999 PMC5123795

[44] Bulfone TC, Malekinejad M, Rutherford GW, Razani N. Outdoor transmission of SARS-CoV-2 and other respiratory viruses: a systematic review. J Infect Dis. 2021;223:550–561. 10.1093/infdis/jiaa742 33249484 PMC7798940

[45] Chia WN, Zhu F, Ong SWX, . Dynamics of SARS-CoV-2 neutralising antibody responses and duration of immunity: a longitudinal study. Lancet Microbe. 2021;2:e240–e249. 10.1016/S2666-5247(21)00025-2 33778792 PMC7987301

[46] Bloise S, Marcellino A, Testa A, . Serum IgG levels in children 6 months after SARS-CoV-2 infection and comparison with adults. Eur J Pediatr. 2021;180:3335–3342. 10.1007/s00431-021-04124-w 34023936 PMC8140562

[47] Seow J, Graham C, Merrick B, . Longitudinal observation and decline of neutralizing antibody responses in the three months following SARS-CoV-2 infection in humans. Nat Microbiol. 2020;5:1598–1607. 10.1038/s41564-020-00813-8 33106674 PMC7610833

[48] Zhu L, Xu X, Zhu B, . Kinetics of SARS-CoV-2 specific and neutralizing antibodies over seven months after symptom onset in COVID-19 patients. Microbiol Spectr. 2021;9:e0059021. 10.1128/Spectrum.00590-21 34550000 PMC8557935

